# The Mobilisation of the Medical Profession

**Published:** 1916-07-01

**Authors:** 


The Hospital

The Workers' Newspaper of Administrative Medicine and Institutional Life, Administration, National Insurance and Health.

No. 1569, Vol. LX. SATURDAY, JULY 1, 1916. PRICE ONE PENNY.

### THE MOBILISATION OF THE MEDICAL PROFESSION.

The letter and memorandum addressed to the members of the medical profession by the Director-General of the Army Medical Service must be welcomed as an authoritative and responsible statement both of the medical needs of the Army and of the manner in which alone these needs can be supplied. The appeal and counsel have more than a personal authority, though this authority in itself has the highest claims to attention. The documents here in question represent without doubt the result of the mature reflections and organised opinions of those who know both the civil and the military necessities of the situation, and they must be accepted as a final and conclusive definition of the actual position. The broad conclusions issuing from these documents are that the Army needs more doctors, that its requirements in this respect cannot be met under the provisions of the Military Service Acts without serious prejudice to the medical wants of the civil population, and that in order to satisfy both the civil and the military aspects of the national situation a further voluntary service is required from the profession. Regarding practitioners under forty-one years of age, the official memorandum adds little to what was stated in these columns in a recent issue. Like their compeers these men are now liable to compulsory military service, and escape is only possible to him who can convince the authorised Tribunal that in his individual case exemption ought to be granted. There is, however, one point emphasised in the memorandum of which, in order to prevent misunderstanding, it is necessary to take note. This refers to the legal position of those practitioners who being of military age enrol with one or other of the authorised professional committees. One advantage of such enrolment, it has been stated, is that it limits the practitioner's term of service to one year, as compared with the non-enrolled practitioner, who is liable to be recruited for the duration of the war. There is here need of qualification. A practitioner who has enrolled and who has served for twelve months in the R.A.M.C. still remains at the end of this period a citizen who is liable for military duty under the Military Service Acts. Hence if the needs of the Army require it he may still be called upon for this duty, and he can only escape the claim by again enrolling with the appropriate professional committee. He may therefore be called upon for a further term of service, though doubtless this summons will only be issued if the Army needs cannot be satisfied by calling to the Colours those who have not previously served. In a word, a twelve months' term in the R.A.M.C. cannot upset the statutory liability to military service created by the compulsory provisions of the Military Service Acts. Yet the advantage rests with the enrolled practitioner as compared with his unenrolled fellow, for the latter has no chance of a term shorter than the "duration of the war," and, further, his pay and allowances will be on a somewhat lower scale than those fixed for men whose names are registered with the professional committees.

To enrol or not to enrol is, however, for the practitioner of military age a personal question. In either event he is under the provisions of the Military Service Acts, and if the Army needs him he has no choice in the matter. When the summons comes he must needs join the Colours. But the Director-General recognises that something more than this is necessary if medical responsibility for the welfare both of His Majesty's Forces and the civil population is to be adequately discharged. To take for the Army a large proportion of the younger men still remaining in civil practice will inevitably mean that particular areas of the country will be deprived of a due amount of medical protection. Especially serious is this consideration in relation to industrial districts, where just now work is being performed of high national importance and under great pressure. To protect and maintain the health of the munition workers is as urgent a claim as is the supply of medical officers to the armies in the field; and, similarly, the general medical care of the civil population is a claim of high necessity. This, in brief, is the position which the profession has now to face. The Director-General has no doubt that it can be faced in one way only, and he does not hesitate to call for the "mobilisation of the whole of the medical services" of the country. What reply has the profession to make to this claim? There is, of course, no legal or compulsory power behind it, but it rests on a moral sanction which few will fail to recognise. The recognition, however, must take the form of action, and this, if it is to have virtue, may not be delayed. Already confirmation of the official definition is heard in the shape of resolutions adopted here and there that, unless the whole of the medical men in the district concerned are mobilised, no more practitioners can be given to the Army without the sacrifice of civil interests. Plainly, the one chance of saving the situation is efficient organisation. The number of doctors is limited, and more cannot be created by stamping on the ground; the work to be covered is now plainly defined; and somehow or other the means must be fitted to the end. The supreme question is whether the medical profession can show itself equal to the occasion. Unless it does so it will certainly come under condemnation. So far it cannot be said that either leadership or organisation has been a conspicuous feature in medical policy. But both the one and the other are imperative in the present crisis. The great majority of the profession are anxious both on patriotic and on professional grounds to give of their best to the nation, and they wait only some commanding voice. The opportunity is a large one, and it cannot be missed without prejudice to interests of commanding importance. Presumably the "professional committees" carry the first responsibility, but it is time these organisations showed themselves alive to the situation. Unless they do so new agencies must be selected, for the profession as a whole stands face to face with a national claim, and a worthy answer is the concern of every one of its members. The time for leadership is here and now, and the hour ought surely to produce the man.

